# Synthesis of Dihydrooxepino[3,2-*c*]Pyrazoles via Claisen Rearrangement and Ring-Closing Metathesis from 4-Allyloxy-1*H*-pyrazoles

**DOI:** 10.3390/molecules23030592

**Published:** 2018-03-06

**Authors:** Yoshihide Usami, Aoi Kohno, Hiroki Yoneyama, Shinya Harusawa

**Affiliations:** Laboratory of Pharmaceutical Organic Chemistry, Osaka University of Pharmaceutical Sciences, 4-20-1 Nasahara, Takatsuki, Osaka 569-1094, Japan; e09627@gap.oups.ac.jp (A.K.); yoneyama@gly.oups.ac.jp (H.Y.); harusawa@gly.oups.ac.jp (S.H.)

**Keywords:** dihydrooxepino[3,2-*c*]pyrazole, synthesis, 4-allyloxy-1*H*-pyrazoles, RCM, Claisen rearrangement

## Abstract

Synthesis of novel pyrazole-fused heterocycles, i.e., dihydro-1*H*- or 2*H*-oxepino[3,2-*c*]pyrazoles (**6** or **7**) from 4-allyloxy-1*H*-pyrazoles (**1**) via combination of Claisen rearrangement and ring-closing metathesis (RCM) has been achieved. A suitable catalyst for the RCM of 5-allyl-4-allyloxy-1*H*-pyrazoles (**4**) was proved to be the Grubbs second generation catalyst (Grubbs^2nd^) to give the predicted RCM product at room temperature in three hours. The same reactions of the regioisomer, 3-allyl-4-allyloxy-1*H*-pyrazoles (**5**), also proceeded to give the corresponding RCM products. On the other hand, microwave aided RCM at 140 °C on both of **4** and **5** afforded mixtures of isomeric products with double bond rearrangement from normal RCM products in spite of remarkable reduction of the reaction time to 10 min.

## 1. Introduction

Because pyrazoles are important heterocyclic compounds with diverse bioactivities, extensive studies have been carried out for the synthesis of substituted or functionalized pyrazoles [1,2,3,4,5]. However, most of them are based on the construction of a pyrazole ring by the [2 + 3] cycloaddition of already substituted parts [6,7,8]. Direct functionalization of pyrazoles has been rarely reported; it is a synthetic challenge. We have been studying the direct functionalization of pyrazoles and reported the synthesis of 4-arylpyrazoles via Kumada–Tamao coupling [9], Suzuki–Miyaura coupling [10], and the Heck–Mizoroki reaction [11]; 2*H*-indazoles via a double Sonogashira coupling followed by Bergmann–Masamune cyclization [12]; and 4-hydroxy-1*H*-pyrazoles by the total synthesis of withasomnine alkaloids as its applications [13,14]. 

**Figure 1:**
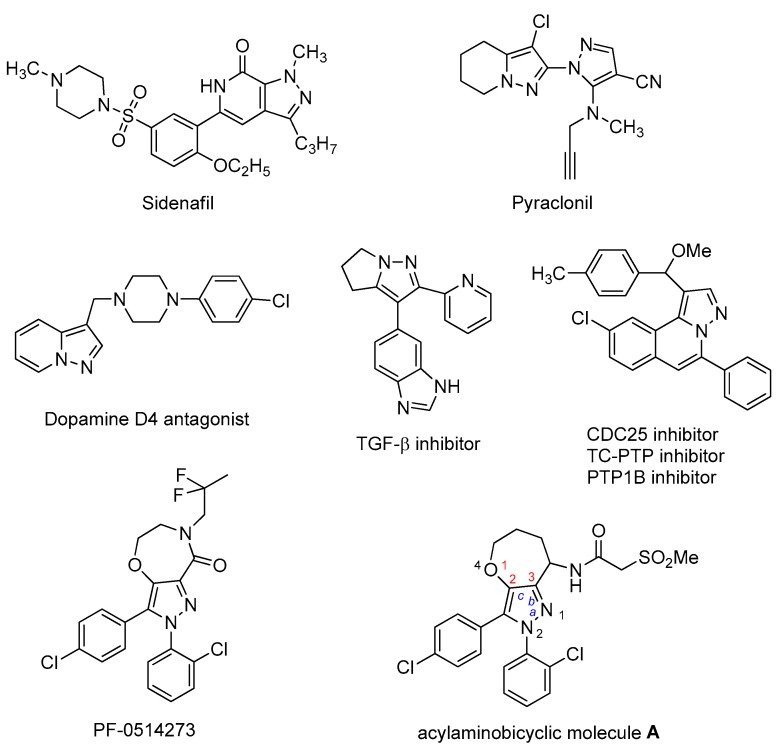
Examples of bioactive pyrazole-fused heterocyclic molecules. TGF-β: Transforming growth factor-β, CDC: Cell Division Cycle (Protein Phosphatase), TC-PTP: T-Cell Protein Tyrosine Phosphatase, PTP1B: Protein tyrosine phosphatase 1B.

On the other hand, pyrazole-fused heterocycles have been recently synthesized because they exhibit diverse important biological activities; they could not be synthesized from substituted monocyclic pyrazole derivatives [15]. Sildenafil citrate, a well-known clinically approved erectile dysfunction improving drug Viagra^®^, is one of the representative example possessing a pyrazole-fused bicyclic structure (Figure 1) [15,16]. Pyraclonil is also well known as an excellent pesticide or herbicide with a similar structural feature [17]. Several examples exhibiting important biological activities are also shown in Figure 1 [18,19,20,21,22,23]. Thus, synthesis of novel pyrazole-fused heterocycles is extremely important for drug discovery and is a great challenge in organic chemistry.

PF-0514273 (Figure 1) is a selective human cannabinoid (hCB) 1 receptor antagonist with a potency of *K*i: 1.8 ± 1.4 nM was developed by a Pfizer research group to reduce the side effect of rimonabant; this compound advanced to human clinical trials for weight management [22]. Furthermore, acylaminobicyclic (**A**), also shown in Figure 1, was evaluated by the same research group; it showed more potent activity as a peripherally targeted hCB1 receptor antagonist (*K*i = 0.54 nM) [23]. Both the molecules have a fused heterocyclic skeleton between a pyrazole and seven-membered ring containing an oxygen atom. Therefore, it is important to develop a new method for the 5,6,7,8-tetrahydrooxepino[3,2-*c*]pyrazole skeleton, which the acylaminobicyclic **A** has.

In our previous synthesis of withasomnines, the key intermediates were 4-allyloxy-1*H*-1-tritylpyrazole (**1a**) and its Claisen rearrangement product, 5-allyl-4-hydroxy-1*H*-1-tritylpyrazole (**2a**) [14]. Compound **2a** or its structural isomer, 3-allyl-4-hydroxy-1*H*-1-trityl pyrazole (**3a**), contains a hydroxyl group, which can be further *O*-functionalized. Recently, the combination of Claisen rearrangement and ring-closing metathesis (RCM) provides a powerful approach to construct various polycyclics [24,25,26,27]. But, examples of synthesis of pyrazole-fused heterocyclic molecules using RCM are rare [28]. Therefore, we attempted to construct pyrazole-fused heterocycles based on **2** or **3**. When **2** or **3** was further *O*-allylated, products **4** and **5** were suitable starting materials for RCM, leading to pyrazole 5,8-dihydro-1*H*-oxepino[3,2-*c*]pyrazoles (**6**) and 5,8-dihydro-2*H*-oxepino[3,2-*c*]pyrazoles (**7**), respectively. Herein, we report the new synthesis of a pyrazole-fused heterocyclic skeleton, dihydrooxepino[3,2-*c*]pyrazoles, from **1** via the combination of Claisen rearrangement and RCM and divergence of RCM products depending on reaction conditions.

## 2. Results and Discussions

### 2.1. Claisen Rearrangement of 4-Allyloxy-1H-pyrazoles in 1,2-Dimethoxyethane

As mentioned in our previous paper, Claisen rearrangement of **1a** in 1,2-dimethoxyethane (DME) showed improved regioselectivity for **2a** (65%): **3a** (1%) compared to the same reaction in *N*,*N*-diethylaniline (DEA) (**2a** (61%): **3a** (3%)) [14]. Another merit of using DME as a solvent is easier purification of the reaction products. DEA must be removed by chromatography, whereas DME can be removed by evaporation. First, the regioselectivity in the Claisen rearrangement of other substrates **1b**–**e** with DME under microwave (MW) irradiation was investigated. The results are summarized in Table 1. The MW reaction conditions were 200 °C and 30 min. In the reaction of substrate **1b** (R = benzyl), 5-allylated product **2b** was obtained exclusively in a similar yield (98%, entry 2) as DEA (**2b**: 92%) reported previously [13,14]. Improved regioselectivity was observed for substrate **1d** bearing an *n*-butyl group, affording 5-allylate **2d** as the sole product in 97% yield (entry 4), whereas a mixture of **2d** (65%) and **3d** (20%) was obtained in the MW reaction with DEA. Surprisingly, reversed regioselectivity was observed in the reaction of substrate **1c** bearing a *p*-toluenesulfonyl substituent at the *N*1 position in DME, giving 3-allylated **3c** (55%, entry 3), whereas **2c** was formed as the major product (65%) and as the minor product (20%) in the MW-assisted Claisen rearrangement in DEA in our previous study [14]. However, we do not have a plausible explanation for this reversed selectivity.

Furthermore, we investigated the relationship between the Claisen rearrangement and the subsequent pattern in the allylic system of substrate **1**, having a substituent at R, R’, and R” positions.

When R’ positions are occupied by methyl group (entries 6 and 9), the Claisen rearrangement did not proceed. Similar results are obtained on the substrates **1e** (R’ = Me) and **1g** (R’ = Ph) having Tr group at R position (entries 5 and 7). Meanwhile, reactions of **1h** (R = Bn, R’ = Me, and R” = H) and **1j** (R = Bn, R’ = Ph, and R” = H) with a benzyl group at the *N*1 position provide rearranged products **2h** and **2j** in 64% and 54% yields, respectively. 

In cases of the substrates with Tr groups (entries 5–7) as well as with a Me group at R” position (entry 9), severe steric repulsions in those transition states may inhibit the Claisen rearrangement. 

From results summarized in Table 1, appearance of the Claisen rearrangement would depend on the substituent pattern in the allylic system of the substrates 1.

**Scheme 1:**
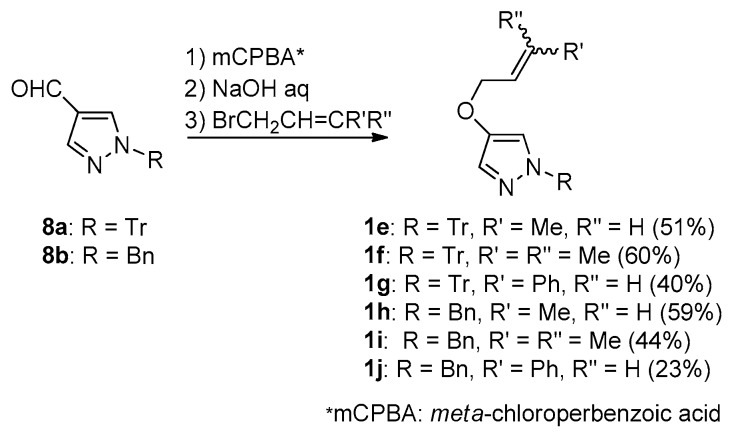
Synthesis of substituted 4-allyloxy-1*H*-pyrazoles.

3′-Substituted 4-allyloxy-1*H*-pyrazoles **1e**–**j** used as substrates were prepared from aldehydes **8a** or **8b**, as illustrated in Scheme 1.

### 2.2. Synthesis of 5- or 3-Allyl-4-allyloxy-1H-pyrazoles

**Scheme 2:**
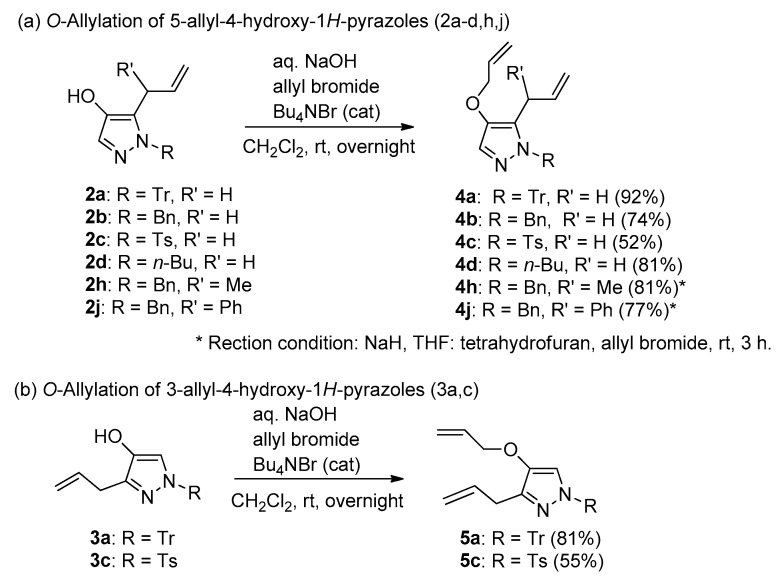
*O*-Allylation of 4-hydroxy-1*H*-pyrazoles (**2a**–**d,h,j** and **3a,c**).

Next, *O*-allylation of the 4-hydroxyl group in **2** or **3** was investigated (Scheme 2). Substrates **2** or **3** reacted with allyl bromide under basic condition at ambient temperature, affording 5-allyl-4-allyloxy-1*H*-pyrazoles (**4a**–**d**,**h**,**j**) and 3-allyl-4-allyloxy-1*H*-pyrazoles (**5a**,**c**) as shown in Scheme 2a,b, respectively. Most of the substrates were *O*-allylated in good yields except **2c** and **3c** bearing a toluenesulfonyl substituent at the *N*1 position. The toluenesulfonyl group at the *N*1 position seems unstable under the basic reaction condition, resulting in a lower yield of **4c** or **5c**. Thus, the RCM substrates for the formation of a seven-membered ring were obtained.

### 2.3. Synthesis of Dihydro-1H-1-Trityloxepino[3,2-c]pyrazoles

Using the prepared substrates, the synthesis of dihydro-1*H*- or 2*H*-oxepino[3,2-*c*]pyrazoles was investigated. First, three types of Grubbs catalysts—Grubbs^1st^, Grubbs^2nd^, and Hoveyda–Grubbs^2nd^—were used in the RCM of substrate **4a** (Table 2). The reaction conditions—solvent, temperature, and amount of ruthenium catalyst—were fixed as CH_2_Cl_2_, room temperature, and 10 mol %, respectively [29,30]. The results are summarized in Table 1. All the reactions afforded the desired 5,8-dihydro-1*H*-1-trityloxepino[3,2-*c*]pyrazole (**6a**), and Grubbs^2nd^ gave the best yield (entry 2, 74% yield). Then, all the following RCMs were carried out using Grubbs^2nd^ as the catalyst.

As described above, the RCM reactions using Grubbs^2nd^ at room temperature took 120–150 min for the complete consumption of starting material **4a**. To shorten the reaction time, next, MW-assisted RCM was investigated. The reaction at a high temperature of 140 °C in CH_2_Cl_2_ as the solvent was achieved using sealed vials as the MW reactor. The results are summarized in Table 3. Interestingly, the ring-closed products with double-bond migration **9a** and **10a** were obtained from substrate **4a** as the major products in various ratios along with a small amount of **6a**, as shown in entries 2–4 [31,32,33,34]. As the best overall yield was obtained in a reaction time of 10 min (entry 4), this condition was applied in the following MW reactions. For reference, the RCM of **4a** at room temperature overnight also provided the isomerized products in a small amount in addition to **6a** as the major product. To complete the isomerization, overnight reflux (40 °C) was required as noted in entry 1 for comparison. Geometries of the double bond of all the products **6a**, **9a**, and **10a** generated in the RCM were assigned as *Z* configuration based on the coupling constant <12 Hz of olefinic protons in their ^1^H-Nucler Magnetic Resonance (NMR) spectra. 

Similarly, the reactions of **4b** (R = Bn) and **4c** (R = Ts) at room temperature gave the desired RCM products **6b** (entry 6) and **6c** (entry 8), respectively, whereas the corresponding MW reactions gave the isomerized products **9b** (25%)/**10b** (63%) (entry 7) and **9c** (21%)/**10c** (50%) (entry 9), respectively. The reaction of **4d** (R = *n*-Bu) proceeded in the same manner, but the reaction product **6d** obtained at room temperature, which was almost pure in the crude ^1^H-NMR spectrum, was partially isomerized to an inseparable mixture of **9d** and **10d** during the purification using a preparative thin layer chromatography (TLC) plate (entry 10). The corresponding MW reaction of **6d** afforded a mixture of **9d** and **10d**, as observed in the ^1^H-NMR spectrum of the crude residue, in 77% combined yield in the ratio ca. 1:3 (entry 11).

The RCM reactions of 3-allylated **5a** and **5c** were also investigated (Table 4). The reaction using Grubbs^2nd^ at room temperature gave only the desired 5,8-dihydro-2*H*-oxepino[3,2-*c*]pyrazoles (**7a** and **7c**) in 75% and 52% yields, respectively (entries 1 and 3). The corresponding MW reaction of **5a** gave isomerized **11a** and **12a** in 13% and 79% yields, respectively (entry 2). The MW reaction of **5c** afforded **11c** (11%) and **12c** (56%) (entry 4).

### 2.4. Double-Bond Migration of Dihydro-1H-1-Trityloxepino[3,2-c]pyrazoles Catalyzed by Ruthenium Hydride Species

**Scheme 3:**
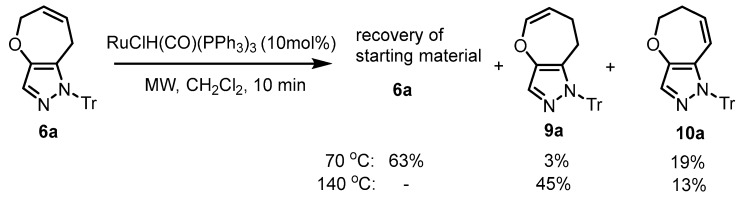
Reaction of **6a** with ruthenium hydride species.

Double-bond migration during the RCM of medium-sized rings has been reported [30,31,32,33]. Previous studies showed the participation of a small amount of ruthenium hydride species present in the used catalyst as the impurity or produced during the RCM process. Then, the reaction of **6a** with carbonylchlorohydrotris(triphenylphosphine)ruthenium (II) (RuClH(CO)(PPh_3_)_3_) was investigated as shown in Scheme 3. The MW reaction at 70 °C for 10 min gave the isomerized products **9a** and **10a** in 3% and 19% yields, respectively, along with the recovery of **6a** (63%), whereas no isomerization was observed in the reaction at 40 °C. The same reaction at 140 °C gave the isomerized products **9a** and **9a** in 45% and 13% yields, respectively. These experiments indicated the participation of ruthenium hydride species in the double-bond isomerization of **6** to **9** or **10** as observed in the RCMs at a higher temperature in this study. 

## 3. Materials and Methods

### 3.1. General

Infrared (IR) spectra were obtained using a Perkin Elmer Fourier Transformation-Infrared (FT-IR) spectrometer 1720X (Perkin Elmer, Walttham, MA, USA). High Resolution Mass Spectra (HRMS) were recorded using a JEOL JMS-700 (2) mass spectrometer (JEOL, Tokyo, Japan). NMR spectra were recorded at 27 °C using Agilent 300-, 400-MR-DD2, and 600-DD2 spectrometers (Agilent Technologies, Santa Clara, CA, USA) in CDCl_3_ using tetramethylsilane (TMS) as the internal standard. Liquid column chromatography was conducted using silica gel FL-60D (Fuji Silysia, Tokyo, Japan). Analytical TLC was performed using precoated plates WAKO silica gel 70 F_254_ (Wako Pure Chemical Industries, Tokyo, Japan) and the compounds were detected by dipping the plates in an ethanol solution of phosphomolybdic acid, followed by heating. Preparative TLC was performed using precoated glass plates silica gel 60 F_254_ (Merck & Co., Inc., Darmstadt, Germany). MW-assisted reactions were carried out using a Biotage Initiator^®^ (Basel, Switzerland). Anhydrous CH_2_CH_2_ was purchased from Wako Pure Chemical Industries (Osaka, Japan). 

### 3.2. O-Allylation of 4-Hydroxy-1H-pyrazoles (Scheme 1)

General procedure: To a solution of 4-formyl-1*H*-1-tritylprazole (**8a**) (94.6 mg, 0.28 mmol) in CH_2_Cl_2_ (6 mL) was added 70% mCPBA (131.8 mg, 0.53 mmol) at 0 °C, with stirring. After 5 h, saturated NaHCO_3_ aq (10 mL) was added to quench the reaction mixture. The mixture was extracted with CH_2_Cl_2_ 3 times. Combined organic layer was dried over MgSO_4_, filtered, and condensed under reduced pressure to give a crude formate. To an acetone solution of the crude formate (6 mL), 20% NaOH aq (4 mL) was added, then the mixture was heated under reflux for 1 h, then crotyl bromide (48 µL, 0.42 mmol) was added to the cooled mixture. After stirring for 3 h, saturated NH_4_Cl aq was added to the reaction mixture to quench, the mixture was condensed under reduced pressure, extracted with CH_2_Cl_2_ for 3 times. The combined CH_2_Cl_2_ layer was dried over MgSO_4_, filtered, and condensed under reduced pressure to give a crude residue, which was purified with flash column chromatography (EtOAc:Hexane = 1:10) to give 4-(2-butenyl)oxy-1*H*-1-tritylpyrazole (**1e**) (54.5 mg, 51%). 

*4-(2-butenyl)oxy-1H-1-tritylpyrazole* (**1e**): white powder; melting point (m.p.) 84–87 °C; IR (KBr) v_max_ 1571 (C=C), 1490 (C=C), 1445 (C=C) cm^−1^; ^1^H-NMR (400 MHz, CDCl_3_): δ 1.63 (0.5 H, dd, *J* = 6.5, 1.0 Hz, (*Z*)-C*H*_3_CH=CH-), 1.71 (2.5 H, dd, *J* = 6.4, 1.0 Hz, (*E*)-C*H*_3_CH=CH-), 4.26 (1.7 H, br d, *J* = 6.4 Hz, (*E*)-CH=CHC*H*_2_O-), 4.41 (0.3 H, dd, *J* = 6.4 Hz, (*Z*)-CH=CHC*H*_2_O-), 5.31–5.63 (1H, m, -C*H*=CH-), 5.63–5.78 (1H, m, -CH=C*H*-), 7.01 (0.83H, d, *J* = 0.8 Hz, pyrazole-H), 7.03 (0.17H, d, *J* = 0.8 Hz, pyrazole-H), 7.10–7.20 (6H, m, Tr-H), 7.22–7.38 (9 H, m, Tr-H), 7.40 (0.83H, d, *J* = 0.6 Hz, pyrazole-H), 7.41 (0.17H, d, *J* = 0.7 Hz, pyrazole-H); ^13^C-NMR (100 MHz, CDCl_3_): δ (13.2), 17.7, 53.4, (67.0), 72.2, 78.5, (81.9), (118.2), 118.3, (125.4), 126.0, 127.2, 128.1, (128.8), 130.0, 130.7, 130.9, 143.1, 144.1, 146.8; High Resolution Electron Impact Mass Spectrum (HREIMS) *m*/*z* calcd. for C_26_H_24_N_2_O [M^+^] 380.1189, found 380.1185.

*4-(3-Methyl-2-butenyl)oxy-1H-1-tritylpyrazole* (**1f**): Colorless crystals (CH_2_Cl_2_); m.p. 60–70 °C; IR (KBr) v_max_ 1576 (C=C), 1492 (C=C), 1445 (C=C) cm^−1^; ^1^H-NMR (600 MHz, CDCl_3_): δ 1.63 (3H, d, *J* = 0.6 Hz, =C*Me*Me), 1.74 (3H, d, *J* = 0.9 Hz, =CMe*Me*), 4.33 (2H, dt, *J* = 7.0 Hz, OC*H*_2_CH=), 5.40 (1H, tqq, *J* = 7.0, 0.9, 0.6 Hz, -OCH_2_C*H*=C(CH_3_)_2_), 7.00 (1H, d, *J* = 0.9 Hz, pyrazole-H), 7.14–7.17 (6H, m, Tr-H), 7.26–7.31 (9 H, m, Tr-H), 7.40 (1H, d, *J* = 0.9 Hz, pyrazole-H); ^13^C-NMR (125 MHz, CDCl_3_): δ 18.1, 25.7, 68.1, 78.5, 118.2, 119.7, 127.6, 127.9, 128.2, 130.1, 138.4, 143.2, 144.2; HREIMS *m*/*z* calcd. for C_28_H_26_N_2_O [M^+^] 394.2045, found 394.2047.

*(E/Z)-4-(3-Phenyl-2-propenyl)oxy-1H-1-tritylpyrazoles* (**1g**): Colorless needles (CH_2_Cl_2_); m.p. 115–120 °C; IR (KBr) v_max_ 1565 (C=C), 1445 (C=C) cm^−1^; ^1^H-NMR (400 MHz, CDCl_3_): δ 4.50 (1.8H, dd, *J* = 6.1, 1.2 Hz, -OC*H*_2_CH=), 4.86 (0.2H, dd, *J* = 6.5, 1.2 Hz, -OC*H*_2_CH=), 6.31 (1H, dt, *J* = 16.0, 6.1 Hz, -CH_2_C*H*=CH-), 6.62 (1H, d, *J* = 16.0 Hz, -CH=C*H*Ph), 7.05 (1H, d, *J* = 0.5 Hz, pyrazole-H), 7.10–7.19 (8H, m, Tr-H, Ph-H), 7.22–7.40 (12H, m, Tr-H, Ph-H), 7.44 (1H, d, *J* = 0.5 Hz, pyrazole-H); ^13^C-NMR (100 MHz, CDCl_3_): δ 29.7, 72.3, 78.7, 118.7, 124.3, 126.6, 127.6, 127.9, 128.3, 128.6, 130.1, 136.3, 143.1, 144.0; HREIMS *m*/*z* calcd. for C_31_H_26_N_2_O [M^+^] 442.2045, found 442.2046.

*(E/Z)-1-Benzyl-4-(2-butenyl)oxy-1H-pyrazoles* (**1h**): Oil; IR (film) v_max_ 1575 (C=C), 1496 (C=C) cm^−1^; ^1^H-NMR (400 MHz, CDCl_3_): δ 1.66 (0.5 H, dd, *J* = 5.8, 0.5 Hz, (*Z*)-C*H*_3_CH=CH-), 1.70 (2.5H, dd, *J* = 6.5, 0.6 Hz, (*E*)-C*H*_3_CH=CH-), 4.28 (1.7 H, dd, *J* = 6.2, 1.0 Hz, (*E*)-OC*H*_2_CH=CH-), 4.42 (0.3 H, dd, *J* = 6.2, 0.6 Hz, (*Z*)-OC*H*_2_CH=CH-), 5.18 (2 H, s, ArC*H*_2_Ph), 5.60–5.72 (1H, m, -CH=C*H*-), 5.72–5.84 (1H, m, -CH=C*H*-), 7.01 (0.83 H, s, pyrazole-H), 7.03 (0.17 H, s, pyrazole-H), 7.17 (2H, dd, *J* = 6.9, 1.1 Hz, Ph-H), 7.20–7.40 (6 H, m, Ph-H, pyrazole-H); ^13^C-NMR (100 MHz, CDCl_3_): δ (13.3), 17.8, (49.7), 56.6, (67.2), 72.4, (114.96), 150.02, (125.5), 126.0, (126.9), 127.5, 127.6, (128.0), (128.3), 128.5, 128.7, (128.88), (128,.92), (129.0), 131.0, 136.7, (143.5), 145.6; HREIMS *m*/*z* calcd. for C_14_H_16_N_2_O [M^+^] 228.1263, found 228.1263.

*1-Benzyl-4-(3-methyl-2-butenyl)oxy-1H-pyrazoles* (**1i**): Oil; IR (film) v_max_ 1574 (C=C), 1496 (C=C), 1455 (C=C) cm^−1^; ^1^H-NMR (400 MHz, CDCl_3_): δ 1.67 (3H, s, =C*Me*Me), 1.74 (3H, s, =CMe*Me*), 4.34 (2H, d, *J* = 6.9 Hz, -OC*H*_2_CH=), 5.18 (2H, s, ArC*H*_2_Ph), 5.42 (1H, m, -CH_2_C*H*=CMe_2_), 7.01 (1H, s, pyrazole-H), 7.18 (2H, d, *J* = 7.3 Hz, Ph-H), 7.24–7.34 (4 H, m, Ph-H, pyrazole-H); ^13^C-NMR (100 MHz, CDCl_3_): δ 18.1, 25.7, 56.6, 68.2, 114.9, 119.6, 127.50, 127.54, 128.0, 128.7, 136.7, 138.6, 145.8; HREIMS *m*/*z* calcd. for C_15_H_18_N_2_O [M^+^] 242.1419, found 242.1420.

*(E/Z)-1-Benzyl-4-(3-phenyl(2-propenyl))oxy-1H-pyrazoles* (**1j**): white powder; m.p. 68–71 °C; IR (KBr) v_max_ 1565 (C=C), 1490 (C=C), 1445 (C=C) cm^−1^; ^1^H-NMR (400 MHz, CDCl_3_): δ 4.50 (1.9H, dd, *J* = 7.1, 1.4 Hz, -OC*H*_2_CH=), 4.58 (0.1H, dd, *J* = 5.8, 1.4 Hz, -OC*H*_2_CH=), 5.17 (1.9H, s, ArC*H*_2_Ph), 5.21 (0.1H, s, ArC*H*_2_Ph), 6.31 (1H, dt, *J* = 16.0, 5.9 Hz, -CH_2_C*H*=CH-), 6.63 (1H, d, *J* = 16.0 Hz, -CH=C*H*Ph), 7.04 (1H, s, pyrazole-H), 7.13–7.36 (11H, m, Ph-H, pyrazole-H); ^13^C-NMR (100 MHz, CDCl_3_): δ 56.7, 72.4, 115.3, 124.3, 126.6, 127.5, 127.7, 127.95, 128.0, 128.6, 128.8, 133.4, 136.3, 136.6, 145.6; HREIMS *m*/*z* calcd. for C_19_H_18_N_2_O [M^+^] 290.1419, found 290.1418.

### 3.3. Claisen Rearrangement of 1-Protected 4-Allyloxy-1H-pyrazoles with DME (Table 1)

*General procedure (Table 1, entry 2)*: A sealed vial containing a solution of **1b** (146.1 mg, 0.68 mmol) in DME (2 mL) was heated at 200 °C for 30 min under MW irradiation. After the cooling, the reaction mixture was quenched with NH_4_Cl aq. and extracted with EtOAc. The organic layer was washed with brine, dried over MgSO_4_, and filtered. The solvent was removed under reduced pressure; the crude residue was subsequently purified by column chromatography (hexane/EtOAc = 3:1 *v*/*v*), affording **2b** (143.2 mg, 98% yield) as an oil.

*3-Allyl-4-allyloxy-1H-1-toluenesulfonylpyrazole* (**3c**); Oil; IR (liquid film) v_max_ 1593 (C=C), 1491 (C=C) cm^−1^; ^1^H-NMR (600 MHz, CDCl_3_): δ 2.41 (3H, s, PhC*H*_3_), 3.68 (2H, dt, *J* = 5.9, 1.5 Hz, ArC*H*_2_CH=CH_2_), 4.41 (2H, dt, *J* = 5.3, 1.5 Hz, -OC*H*_2_CH=CH_2_), 5.01 (1H, dq, *J* = 17.0, 1.4 Hz, -CH=CH*H*), 5.06 (1H, dq, *J* = 10.0, 1.5 Hz, -CH=CH*H*), 5.25 (1H, dq, *J* = 10.6, 1.4 Hz, -CH=CH*H*), 5.32 (1H, dq, *J* = 17.3, 1.5 Hz, -CH=C*H*H), 5.89–5.97 (2H, m, ArCH_2_C*H*=CH_2_), 5.91 (1H, m, -CH_2_C*H*=CH_2_), 7.29 (2H, br d, *J* = 8.5 Hz, Ph-H), 7.52 (1H, s, pyrazole-H), 7.83 (2H, br d, *J* = 8.5 Hz, Ph-H); ^13^C-NMR (125 MHz, CDCl_3_): δ 21.7, 27.8, 73.1, 116.6, 118.3, 128.0, 129.8, 130.7, 132.8, 133.8, 134.4, 134.8, 143.7, 145.4; HREIMS *m*/*z* calcd. for C_16_H_18_N_2_O_3_S [M^+^] 318.1038, Found 318.1035.

*1-Benzyl-4-hydroxy-5-(1-methyl-2-propenyl)-1H-pyrazoles* (**2h**): White powder; m.p. 95–100 °C; IR (KBr) v_max_ 1497 (OH), 1591 (C=C), 1455 (C=C) cm^−1^; ^1^H-NMR (400 MHz, CDCl_3_): δ 1.25 (3H, d, *J* = 7.2 Hz, CH_2_CH-), 3.51 (1H, m, ArC*H*CH_3_CH=), 4.99 (1H, br d, *J* = 17. 4 Hz, -CH=CH*H*), 5.06 (1H, br d, *J* = 10.4 Hz, -CH=CH*H*), 5.21 (1H, d, *J* = 17.2 Hz, ArC*HA*H_B_Ph), 5.25 (1H, d, *J* = 17.2 Hz, ArCH*AH*_B_Ph), 5.96 (1H, ddd, *J* = 17.2, 10.2, 5.5 Hz, -CHC*H*=CH_2_), 7.03 (2H, d, *J* = 6.8 Hz, Ph-H), 7.18 (1H, s, pyrazole-H), 6.00 (1H, ddt, *J* = 17.2, 10.5, 5.3 Hz, -OCH_2_C*H*=CH_2_), 7.25–7.31 (3H, m, Ph-H); ^13^C-NMR (100 MHz, CDCl_3_): δ 17.6, 33.6, 53.9, 114.5, 126.5, 127.6, 128.5, 128.6, 129.9, 137.4, 138.6, 139.2; HREIMS *m*/*z* calcd. for C_14_H_16_N_2_O [M^+^] 228.1263, found 228.1262.

*1-Benzyl-4-hydroxy-5-(1-phenyl-2-propenyl)-1H-pyrazoles* (**2j**): Powder; m.p. 88–93 °C; IR (KBr) v_max_ 3458 (OH), 1591 (C=C), 1496 (C=C), 1455 (C=C) cm^−1^; ^1^H-NMR (400 MHz, CDCl_3_): δ 4.68 (1H, d, *J* = 6.6 Hz, ArC*H*PhCH=), 4.89 (1H, dt, *J* = 17.2, 1.4 Hz, -CH=CH*H*), 5.06 (1H, d, *J* = 16.2 Hz, ArC*HA*H_B_Ph), 5.15 (1H, d, *J* = 16.2 Hz, ArCH*AH*_B_Ph), 5.18 (1H, d, *J* = 10.3 Hz, -CH=CH*H*), 5.29 (1H, ddd, *J* = 17.0, 10.2, 6.8 Hz, -CHC*H*=CH_2_), 6.98 (2H, br d, *J* = 6.7 Hz, Ph-H), 7.10 (2H, br d, *J* = 6.9 Hz, Ph-H), 7.11–7.28 (8H, m, Ph-H, pyrazole-H); ^13^C-NMR (100 MHz, CDCl_3_): δ 45.2, 54.1, 17.4, 126.6, 127.1, 127.6, 127.9, 128.5, 128.7, 128.8, 136.8, 137.0, 139.4, 139.7; HREIMS *m*/*z* calcd. for C_19_H_18_N_2_O [M^+^] 290.1419, found 290.1415.

### 3.4. O-Allylation of 1-Protected 5- or 3-Allyl-4-allyloxy-1H-pyrazoles (Scheme 2)

General procedure: To a solution of 5-allyl-4-hydroxy-1-trityl-1*H*-pyrazole (**2a**) (0.21 g, 0.56 mmol) in CH_2_Cl_2_ (4 mL), 20% NaOH aq. (3 mL) and allyl bromide (120 µL, 1.4 mmol) were added. After stirring at room temperature (r.t.) overnight, the reaction mixture was quenched with sat. NH_4_Cl aq., and then extracted with CH_2_Cl_2_. The organic layer was dried over anhydrous MgSO_4_, filtered, and evaporated. The crude residue was purified using flash chromatography (eluent:hexane/EtOAc gradient), affording **4a** (0.21 g, 92% yield). 

*5-Allyl-4-allyloxy-1-trityl-1H-pyrazole* (**4a**): Colorless crystals (CH_2_Cl_2_); m.p. 110–114 °C; IR (film) v_max_ 1578 (C=C), 1492 (C=C), 1445 (C=C) cm^−1^; ^1^H-NMR (500 MHz, CDCl_3_): δ 2.85 (2H, br dt, *J* = 6.6, 1.1 Hz, ArC*H*_2_CH=CH_2_), 4.43 (2H, dt, *J* = 5.3, 1.6 Hz, OC*H*_2_CH=CH_2_), 4.62 (1H, ddd, *J* = 17.2, 3.2, 1.4 Hz, -CH=CH*H*), 4.65 (1H, ddd, *J* = 10.1, 3.2, 1.3 Hz, -CH=CH*H*), 5.00 (1H, ddt, *J* = 17.2, 10.1, 6.6 Hz, ArC*H*=CH_2_), 5.23 (1H, ddt, *J* = 10.5, 3.0, 1.4 Hz, -CH_2_CH=C*H*H), 5.36 (1H, dq, *J* = 17.2, 1.6 Hz, -CH_2_CH=CH*H*), 6.00 (1H, ddt, *J* = 17.2, 10.5, 5.3 Hz, -OCH_2_C*H*=CH_2_), 7.10–7.14 (6H, m, Tr-H), 7.23–7.31 (9 H, m, Tr-H), 7.33 (1H, s, pyrazole-H); ^13^C-NMR (125 MHz, CDCl_3_): δ 31.2, 72.4, 78.5, 115.6, 117.2, 125.3, 125.4, 127.5, 129.1, 130.1, 132.5, 133.7, 143.0, 144.2; HREIMS *m*/*z* calcd. for C_28_H_26_N_2_O [M^+^] 406.2045, found 406.2049. 

*5-Allyl-4-allyloxy-1H-1-benzylpyrazole* (**4b**): Oil; IR (film) v_max_ 1580 (C=C), 1492 (C=C), 1408 (C=C) cm^−1^; ^1^H-NMR (600 MHz, CDCl_3_): δ 3.26 (2H, br dt, *J* = 5.8, 1.6 Hz, ArC*H*_2_CH=CH_2_), 4.43 (2H, dt, *J* = 5.6, 1.5 Hz, -OC*H*_2_CH=CH_2_), 4.97 (1H, ddd, *J* = 17.0, 3.2, 1.7 Hz, -CH=CH*H*), 5.04 (1H, ddd, *J* = 10.3, 3.3, 1.5 Hz, -CH=CH*H*), 5.21 (2H, s, PhC*H*_2_O-), 5.24 (1H, ddd, *J* = 10.2, 3.0, 1.5 Hz, -CH=C*H*H), 5.35 (1H, ddd, *J* = 17.0, 3.3, 1.5 Hz, -CH=C*H*H), 5.77 (1H, ddt, *J* = 17.0, 10.5, 5.8 Hz, ArCH_2_C*H*=CH_2_), 6.01 (1H, ddt, *J* = 17.3, 10.5, 5.3 Hz, -OCH_2_C*H*=CH_2_), 7.15 (2H, br d, *J* = 7.4 Hz, Ph-H), 7.25 (1H, br t, *J* = 7.4 Hz, Ph-H), 7.29 (2H, br t, *J* = 7.4 Hz, Ph-H), 7.31 (1H, s, pyrazole-H); ^13^C-NMR (150 MHz, CDCl_3_): δ 27.1, 53.8, 73.3, 116.3, 117.6, 126.7, 126.9, 127.0, 127.5, 128.6, 133.69, 133.71, 137.1,142.3; HREIMS *m*/*z* calcd. for C_16_H_18_N_2_O [M^+^] 254.1419, found 254.1416. 

*5-Allyl-4-allyloxy-1H-1-toluenesulfonylpyrazole* (**4c**): Oil; IR (liquid film) v_max_ 1593 (C=C), 1491 (C=C) cm^−1^; ^1^H-NMR (600 MHz, CDCl_3_): δ 2.41 (3H, s, PhC*H*_3_), 3.68 (2H, dt, *J* = 5.9, 1.5 Hz, ArC*H*_2_CH=CH_2_), 4.41 (2H, dt, *J* = 5.3, 1.5 Hz, -OC*H*_2_CH=CH_2_), 5.01 (1H, dq, *J* = 17.0, 1.4 Hz, -CH=CH*H*), 5.06 (1H, dq, *J* = 10.0, 1.5 Hz, -CH=CH*H*), 5.25 (1H, dq, *J* = 10.6, 1.4 Hz, -CH=CH*H*), 5.32 (1H, dq, *J* = 17.3, 1.5 Hz, -CH=C*H*H), 5.89–5.97 (2H, m, ArCH_2_C*H*=CH_2_), 5.91 (1H, m, -CH_2_C*H*=CH_2_), 7.29 (2H, br d, *J* = 8.5 Hz, Ph-H), 7.52 (1H, s, pyrazole-H), 7.83 (2H, br d, *J* = 8.5 Hz, Ph-H); ^13^C-NMR (125 MHz, CDCl_3_): δ 21.7, 27.8, 73.1, 116.6, 118.3, 128.0, 129.8, 130.7, 132.8, 133.8, 134.4, 134.8, 143.7, 145.4; HREIMS *m*/*z* calcd. for C_16_H_18_N_2_O_3_S [M^+^] 318.1038, found 318.1035. 

*5-Allyl-4-allyloxy-1-butyl-1H-pyrazole* (**4d**): Oil; IR (film) v_max_ 1588 (C=C), 1413 (C=C) cm^−1^; ^1^H-NMR (600 MHz, CDCl_3_): δ 0.92 (3H, t, *J* = 7.3 Hz, -CH_2_C*H*_3_), 1.32 (2H, m, -CH_2_C*H*_2_CH_3_), 1.32 (2H, m, -CH_2_C*H*_2_CH_3_), 1.76 (2H, m, -CH_2_C*H*_2_CH_2_-), 3.37 (2H, dt, *J* = 5.6, 1.5 Hz, ArC*H*_2_CH=CH_2_), 3.93 (2H, br t, *J* = 7.3 Hz, ArC*H*_2_CH_2_-), 4.41 (2H, dt, *J* = 5.9, 1.8 Hz, OC*H*_2_CH=CH_2_), 5.01 (1H, dq, *J* = 17.0, 1.8 Hz, -CH=CH*H*), 5.09 (1H, dq, *J* = 10.0, 1.5 Hz, -CH=CH*H*), 5.24 (1H, dq, *J* = 10.6, 1.5 Hz, -CH=CH*H*), 5.35 (1H, dq, *J* = 17.4, 1.8 Hz, -CH=C*H*H), 5.86 (1H, ddt, *J* = 17.0, 10.6, 5.6 Hz, ArCH_2_C*H*=CH_2_), 6.01 (1H, ddt, *J* = 17.4, 10.0, 5.9 Hz, -OCH_2_C*H*=CH_2_), 7.23 (1H, s, pyrazole-H); ^13^C-NMR (150 MHz, CDCl_3_): δ 13.7, 19.9, 27.1, 32.2, 49.5, 73.3, 116.2, 117.6, 126.3, 133.9, 134.1; HREIMS *m*/*z* calcd. for C_13_H_20_N_2_O [M]^+^ 220.1575, found 220.1575. 

*3-Allyl-4-allyloxy-1H-1-tritylpyrazole* (**5a**): Colorless needles (CH_2_Cl_2_/hexane); m.p. 62–65 °C; IR (KBr) v_max_ 1568 (C=C), 1488 (C=C), 1442 (C=C) cm^−1^; ^1^H-NMR (600 MHz, CDCl_3_) δ 3.38 (2H, dt, *J* = 6.2, 1.7 Hz, ArC*H*_2_CH=CH_2_), 4.24 (2H, dt, *J* = 5.6, 1.5 Hz, -OC*H*_2_CH=CH_2_), 4.99 (1H, dq, *J* = 10.0, 1.7 Hz, ArCH_2_CH=C*H*_A_H_B_), 5.03 (1H, dq, *J* = 17.0, 1.7 Hz, ArCH_2_CH=CH_A_*H*_B_), 5.18 (1H, dq, *J* = 10.5, 1.4 Hz, -OCH_2_CH=C*H*_A_H_B_), 5.26 (1H, dq, *J* = 17.3, 1.7 Hz, -OCH_2_CH=CH_A_*H*_B_), 5.93 (1H, ddt, *J* = 17.3, 10.4, 5.6 Hz, -OCH_2_C*H*=CH_2_), 5.99 (1H, ddt, *J* = 17.0, 10.0, 6.2 Hz, ArCH_2_C*H*=CH_2_), 6.88 (1H, s, pyr-H), 7.15–7.19 (6H, m, Tr-H), 7.26–7.30 (9H, m Tr-H); ^13^C-NMR (125 MHz, CDCl_3_): δ 30.0, 72.9, 78.1, 115.1, 117.4, 118.5, 127.4, 127.6, 130.1, 133.5, 135.8, 140.1, 141.6, 143.5; HREIMS *m*/*z* calcd. for C_28_H_26_N_2_O [M^+^] 406.2045, found 406.2043.

*3-Allyl-4-allyloxy-1-toluenesulfonyl-1H-pyrazole* (**5c**): Colorless needles (CH_2_Cl_2_/hexane); m.p. 82–84 °C; IR (KBr) v_max_ 1540 (C=C), 1500 (C=C) cm^−1^; ^1^H-NMR (600 MHz, CDCl_3_) δ 2.41 (3H, s, ArC*H*_3_), 3.33 (2H, dt, *J* = 6.5, 1.7 Hz, ArC*H*_2_CH=CH_2_), 4.38 (2H, dt, *J* = 5.3, 1.5 Hz, -OC*H*_2_CH=CH_2_), 5.00 (1H, dq, *J* = 8.8, 1.5 Hz, -CH=CH*H*), 5.02–5.03 (1H, m, -CH=CH*H*), 5.29 (1H, dq, *J* = 10.5, 1.5 Hz, -CH=CH*H*), 5.37 (1H, dq, *J* = 17.3, 1.5 Hz, -CH=C*H*H), 5.93–5.98 (2H, m, 2 × -CH_2_C*H*=CH_2_), 7.28 (2H, d, *J* = 7.5 Hz, Ts-H), 7.51 (1H, s, pyr-H), 7.81 (2H, d, *J* = 7.5 Hz, Ts-H); ^13^C-NMR (150 MHz, CDCl_3_) δ 22.7, 30.1, 72.3, 113.5, 116.7, 118.3, 127.7, 129.8, 132.2, 133.3, 134.3, 145.2, 145.5, 148.9; HREIMS *m*/*z* calcd. for C_16_H_18_N_2_O_3_S [M]^+^ 318.1038, found 318.1041.

General procedure under aprotic condition: To a solution of **2h** (57.7 mg, 0.27 mmol) in dry THF (2 mL), 60%NaH (16.0 mg, 0.40 mmol) was added at rt. 20 Min later, allyl bromide (34 µL, 0.40 mmol) was added to the reaction flask. After stirring at rt for 3 h, the reaction mixture was quenched with sat. NH_4_Cl aq., then extracted with CH_2_Cl_2_. The organic layer was dried over anhydrous MgSO_4_, filtered, and evaporated. The crude residue was purified using column chromatography (eluent:hexane/EtOAc = 3:1), affording **4h** as a colorless oil (49.9 mg, 81% yield). 

*5-(1-methyl-2-propenyl)-4-allyloxy-1-benzyk-1H-pyrazole* (**4h**): Oil; IR (film) v_max_ 1575 (C=C), 1496 (C=C), 1456 (C=C) cm^−1^; ^1^H-NMR (400 MHz, CDCl_3_): δ 1.28 (3H, d, *J* = 7.3 Hz, -CHC*H*_3_), 3.49 (1H, br quint, *J* = 7.3 Hz, ArC*H* (CH_3_)CH=), 4.41 (2H, br d, *J* = 4.9 Hz, -OC*H*_2_CH=CH_2_), 4.86 (1H, dt, *J* = 17.2, 1.4 Hz, -CHCH=CH*H*), 4.94 (1H, dt, *J* = 10.2, 1.4 Hz, -CHCH=CH*H*), 5.20 (1H, br d, *J* = 16.2 Hz, ArC*H*_A_H_B_Ph), 5.22 (1H, dq, *J* = 10.4, 1.4 Hz, -CH_2_CH=CH*H*), 5.23 (1H, br d, *J* = 16.2 Hz, ArCH_A_*H*_B_Ph), 5.36 (1H, dq, *J* = 17.2, 1.4 Hz, -CH_2_CH=CH*H*), 5.80–6.06 (2H, m (overlapped), 2 × -C*H*=CH_2_), 7.02 (2H, br d, *J* = 6.7 Hz, Ph-H), 7.23–7.31 (4 H, m, Ph-H, pyrazole-H); ^13^C-NMR (100 MHz, CDCl_3_): δ 18.1, 34.3, 54.1, 72.8, 113.9, 117.2, 126.5, 126.9, 127.5, 128.6, 131.0, 133.7, 137.5, 139.6, 142.2; HREIMS *m*/*z* calcd. for C_17_H_20_N_2_O [M^+^] 268.1576, found 268.1579.

*5-(1-phenyl-2-propenyl)-4-allyloxy-1-benzyk-1H-pyrazole* (**4j**): Oil; IR (film) v_max_ 1576 (C=C), 1496 (C=C) cm^−1^; ^1^H-NMR (400 MHz, CDCl_3_): δ 4.33 (1H, br dd, *J* = 11.5, 4.5 Hz, -OC*H*_A_H_B_CH= CH_2_), 4.36 (1H, br dd, *J* = 11.5, 4.5 Hz, -OCH_A_*H*_B_CH= CH_2_), 4.68 (1H, d, *J* = 7.4 Hz, ArC*H*PhCH=), 4.86 (1H, br d, *J* = 17.0 Hz, -CH=C*H*H), 5.06–5.14 (3H, overlapped, 2 × benzyl methylene, -CH=C*H*), , 5.20 (1H, br dq, *J* = 10.4, 1.4 Hz, -CH_2_CH=C*H*H), 5.29 (1H, br dq, *J* = 17.2, 1.5 Hz, -CH_2_CH=CH*H*), 5.91 (1H, ddt, *J* = 15.8, 10.0, 4.7 Hz, -OCH_2_C*H*=CH_2_), 6.30 (1H, ddd, *J* = 17.0, 9.4, 7.9 Hz, -CH_2_C*H*=CH_2_), 5.80–6.06 (2H, m (overlapped), 2 × -C*H*=CH_2_), 6.96 (2H, br d, *J* = 7.1 Hz, Ph-H), 7.09 (2H, br d, *J* = 7.2 Hz, Ph-H), 7.15–7.27 (6H, m, Ph-H), 7.33 (1 H, s, pyrazole-H); ^13^C-NMR (100 MHz, CDCl_3_): δ 45.7, 54.3, 73.0, 116.3, 117.3, 126.7, 127.3, 127.6, 127.9, 128.5, 128.6, 129.6, 133.7, 136.9, 137.1, 140.4, 142.7; HREIMS *m*/*z* calcd. for C_22_H_22_N_2_O [M^+^] 330.1732, found 330.1732.

### 3.5. RCM of 1-Protected 4-Allyloxy- 5- or 3-Allyl-1H-pyrazoles (Table 1, Table 2 and Table 3)

General procedure for the reactions at room temperature (Table 2, entry 2): To a solution of **4a** (18.5 mg, 0.046 mmol) in CH_2_Cl_2_ (4 mL) was added Grubbs^2nd^ (3.9 mg, 0.0046 mmol) under argon atmosphere. After stirring for 120 min, the solvent was removed under reduced pressure. The crude residue was purified by preparative TLC (eluent: hexane/AcOEt = 5:1 *v*/*v*), affording **6a** (12. 7 mg, 74% yield). 

General procedure for the MW-assisted reactions (Table 3, entry 5): To a solution of **4a** (18.5 mg, 0.046 mmol) in CH_2_Cl_2_ (4 mL) in a MW reactor vial was added Grubbs^2nd^ (4.2 mg, 0.0049 mmol, 10 mol %) under argon atmosphere. The reaction vial was irradiated at 140 °C for 120 min. After cooling the reaction mixture, the solvent was removed under reduced pressure. The crude residue was purified by preparative TLC (eluent: hexane/AcOEt = 5:1 *v*/*v*), affording **6a** (0.9 mg, 5% yield), **9a** (7.5 mg, 41% yield), and **10a** (5.7 mg, 33% yield).

*5,8-Dihydro-1H-1-trityloxepino[3,2-c]pyrazole* (**6a**): Colorless crystals (CH_2_Cl_2_/hexane); m.p. 92–95 °C; IR (KBr) v_max_ 1584 (C=C), 1492 (C=C), 1445 (C=C) cm^−1^; ^1^H-NMR (600 MHz, CDCl_3_) δ 2.63 (2H, m, ArC*H*_2_CH=), 4.45 (2H, m, -OC*H*_2_CH_2_=), 5.40 (1H, dtt, *J* = 11.8, 5.0, 1.4 Hz, -CH=C*H*CH_2_Ar), 5.68 (1H, dtt, *J* = 11.8, 5.0, 1.8 Hz, -OCH_2_C*H*=CH-), 7.10–7.13 (6H, m, Tr-H), 7.26–7.32 (10H, m, Tr-H, py-H); ^13^C-NMR (150 MHz, CDCl_3_) δ 27.3, 68.2, 78.5, 126.2, 127.4, 127.5, 127.6, 127.9, 128.3, 129.96, 130.03, 130.7, 142.8, 144.9; HREIMS *m*/*z* calcd. for C_26_H_22_N_2_O [M]^+^ 378.1732, found 378.1730.

*7,8-Dihydro-1H-1-trityloxepino[3,2-c]pyrazole* (**9a**): Colorless crystals (CH_2_Cl_2_/hexane); m.p. 154–158 °C; IR (KBr) v_max_ 1658 (C=C), 1579 (C=C), 1492 (C=C), 1447 (C=C) cm^−1^; ^1^H-NMR (600 MHz, CDCl_3_) δ 1.74 (2H, m, =CHC*H*_2_CH_2_-), 2.25 (2H, m, -CH_2_C*H*_2_Ar), 4.60 (1H, dt, *J* = 7.6, 5.6 Hz, -CH=C*H*CH_2_-), 6.19 (1H, dt, *J* = 7.6, 1.5 Hz, -OC*H*=CHCH_2_-), 7.12–7.15 (6H, m, Tr-H), 7.25–7.32 (10H, m, Tr-H, pyr-H); ^13^C-NMR (150 MHz, CDCl_3_) δ 23.5, 27.9, 78.5, 105.7, 127.3, 127.6, 127.9, 129.9, 131.3, 141.7, 142.2, 143.1; HREIMS *m*/*z* calcd. for C_26_H_23_N_2_O [M + H]^+^ 379.1810, found 379.1813.

*5,6-Dihydro-1H-oxepino[3,2-c]pyrazole* (**10a**): Colorless crystals (CH_2_Cl_2_/hexane); m.p. 144–148 °C; IR (KBr) v_max_ 1564 (C=C), 1489 (C=C), 1446 (C=C) cm^−1^; ^1^H-NMR (600 MHz, CDCl_3_) δ 2.54 (2H, m, =CHC*H*_2_CH_2_-), 4.08 (2H, m, -OC*H*_2_CH_2_-), 5.30 (1H, dt, *J* = 11.5, 5.2 Hz, -CH=C*H*CH_2_-), 5.70 (1H, br d, *J* = 11.5 Hz, ArC*H*=CHCH_2_-), 7.12–7.15 (6H, m, Tr-H), 7.25–7.32 (10H, m, Tr-H, py-H); ^13^C-NMR (150 MHz, CDCl_3_) δ 33.8, 68.8, 78.9, 119.5, 126.3, 127.28, 127.33, 127.5, 128.9, 130.0, 143.1, 145.9; HREIMS *m*/*z* calcd. for C_26_H_22_N_2_O [M]^+^ 378.1732, found 378.1736.

*1-Benzyl-5,8-dihydro-1H-oxepino[3,2-c]pyrazole* (**6b**): Oil; IR (film) v_max_ 1654 (C=C) cm^−1^; ^1^H-NMR (600 MHz, CDCl_3_) δ 3.38 (2H, m, ArC*H*_2_CH=), 4.45 (2H, dq, *J* = 5.5, 1.2 Hz, -OC*H*_2_CH=CHCH_2_-), 5.20 (2H, s, NC*H*_2_Ph), 5.80 (1H, m, -CH=C*H*CH_2_-), 6.28 (1H, m, -CH=C*H*CH_2_-), 7.06 (2H, br d, *J* = 7.3 Hz, Ph-H), 7.25 (1H, s, pyr-H), 7.26 (1H, br t, *J* = 7.3 Hz, Ph-H), 7.33 (1H, br t, *J* = 7.3 Hz, Ph-H); ^13^C-NMR (150 MHz, CDCl_3_) δ 25.9, 29.7, 53.9, 68.2, 126.6, 126.8, 127.7128.5, 128.8, 129.0, 136.9, 144.6; HREIMS *m*/*z* calcd. for C_14_H_14_N_2_O [M]^+^ 226.1106, found 226.1105.

*1-Benzyl-7,8-dihydro-1H-oxepino[3,2-c]pyrazole* (**9b**): Oil; IR (liquid film) v_max_ 1652 (C=C), 1583 (C=C) cm^−1^; ^1^H-NMR (600 MHz, CDCl_3_) δ 2.33 (2H, m, -=CHC*H*_2_CH_2_-), 2.75 (2H, m, -CH_2_C*H*_2_Ar), 4.85 (1H, dt, *J* = 7.4, 6.4 Hz, -CH=C*H*CH_2_-), 5.24 (2H, s, -C*H*_2_Ph), 6.30 (1H, dt, *J* = 7.4, 1.1 Hz, -OC*H*=CHCH_2_-), 7.25 (2H, br d, *J* = 7.7 Hz, Ph-H), 7.26 (1H, br t, *J* = 7.4 Hz, Ph-H), 7.27 (1H, s, pyr-H), 7.31 (2H, br t, *J* = 7.7 Hz, Ph-H); ^13^C-NMR (150 MHz, CDCl_3_) δ 23.2, 24.9, 54.1, 106.8, 126.4, 127.6, 128.1 128.4, 128.8, 137.2, 140.7, 143.3; HREIMS *m*/*z* calcd. for C_14_H_15_N_2_O [M + H]^+^ 227.1184, found 227.1182.

*1-Benzyl-5,6-dihydro-1H-oxepino[3,2-c]pyrazole* (**10b**): Oil; IR (liquid film) v_max_ 1564 (C=C), 1496 (C=C) cm^−1^; ^1^H-NMR (600 MHz, CDCl_3_) δ 2.67 (2H, m, -=CHC*H*_2_CH_2_-), 4.13 (2H, m, -CH_2_C*H*_2_O-), 5.30 (2H, br s, NC*H*_2_Ph), 5.80 (1H, dt, *J* = 11.1, 5.3 Hz, -CH=C*H*CH_2_-), 6.28 (1H, br d, *J* = 11.1 Hz, ArC*H*=CH-), 7.11 (2H, br d, *J* = 7.0 Hz, Ph-H), 7.22 (1H, d, *J* = 0.5 Hz, pyr-H), 7.26 (1H, br t, *J* = 7.0 Hz, Ph-H), 7.31 (2H, br t, *J* = 7.0 Hz, Ph-H); ^13^C-NMR (150 MHz, CDCl_3_) δ 34.2, 53.9, 68.5, 116.4, 126.5, 126.9, 127.6, 127.7, 128.7, 129.0, 137.2, 144.8; HREIMS *m*/*z* calcd. for C_14_H_15_N_2_O [M + H]^+^ 227.1184, found 227.1184.

*5,8-Dihydro-1-toluenesulfonyl-1H-oxepino[3,2-c]pyrazole* (**6c**): Oil; IR (liquid film) v_max_ 1595 (C=C) cm^−1^; ^1^H-NMR (400 MHz, CDCl_3_) δ 2.42 (3H, s, ArC*H*_3_), 3.93 (2H, m, -OC*H*_2_CH=), 4.42 (2H, d, *J* = 4.3 Hz, =CHC*H*_2_Ar), 5.94–5.95 (2H, m, -OCH_2_CH=C*H*CH_2_Ar, -OCH_2_C*H*=CHCH_2_Ar), 7.32 (2H, d, *J* = 8.0 Hz, Ph-H), 7.44 (1H, s, pyr-H), 7.81 (2H, d, *J* = 8.4 Hz, Ph-H); ^13^C-NMR (100 MHz, CDCl_3_) δ 21.7, 26.5, 67.7, 118.5, 127.6, 127.7, 128.4, 130.0, 134.7, 136.1, 137.5, 145.5; HREIMS *m*/*z* calcd. for C_14_H_14_N_2_O [M]^+^ 226.1106, found 226.1105. 

*7,8-Dihydro-1H-oxepino[3,2-c]-1-tolenesulfonylpyrazole* (**9c**): Oil; IR (film) v_max_ 1579 (C=C), 1480 (C=C) cm^−1^; ^1^H-NMR (600 MHz, CDCl_3_) δ 2.39–2.43 (2H, m, =CHC*H*_2_CH_2_-), 2.42 (3H, s, C*H*_3_-Ar), 3.26–3.29 (2H, m, ArC*H*_2_CH_2_-), 5.06 ( 1H, q, *J* = 6.7 Hz, -CH=C*H*CH_2_-), 6.29 (1H, br d, *J* = 7.0 Hz, -OC*H*=CH-), 7.31 (2H, br d, *J* = 8.0 Hz, Ph-H), 7.48 (1H, s, pyr-H), 7.69 (2H, br d, *J* = 8.0 Hz, Ph-H); ^13^C-NMR (150 MHz, CDCl_3_) δ 21.7, 33.8, 68.8, 116.9, 127.7, 128.7, 129.8, 130.1, 131.7, 134.7, 136.7, 145.3, 146.5; HREIMS *m*/*z* calcd. for C_14_H_15_N_2_O_3_S [M]^+^ 290.0725, found 290.0725.

*5,6-Dihydro-1-toluenesulfonyl-1H-oxepino[3,2-c]pyrazole* (**10c**): Oil; IR (film) v_max_ 1579 (C=C), 1480 (C=C) cm^−1^; ^1^H-NMR (600 MHz, CDCl_3_) δ 2.41 (3H, s, C*H*_3_-Ar), 2.66–2.70 (2H, m, =CHC*H*_2_CH_2_-), 4.09–4.13 (2H, m, -OC*H*_2_CH_2_-), 6.05 (1H, dt, *J* = 9.8, 5.3 Hz, -CH=C*H*CH_2_-), 7.24 (1H, br d, *J* = 10.0 Hz, ArC*H*=CH-), 7.30 (2H, br d, *J* = 8.2 Hz, Ph-H), 7.44 (1H, d, *J* = 0.6 Hz, pyr-H), 7.80 (2H, br d, *J* = 8.2 Hz, Ph-H); ^13^C-NMR (150 MHz, CDCl_3_) δ 21.7, 33.8, 68.8, 116.9, 127.7, 128.7, 129.8, 130.1, 131.7, 134.7, 136.7, 145.3, 146.5; HREIMS *m*/*z* calcd. for C_14_H_15_N_2_O_3_S [M]^+^ 290.0725, found 290.0724. 

*1-n-Butyl-5,8-dihydro-1H-oxepino[3,2-c]pyrazole* (**6d**): Oil; IR (film) v_max_ 1658 (C=C) cm^−1^; ^1^H-NMR (400 MHz, CDCl_3_) δ 0.91 (3H, t, *J* = 7.4 Hz, C*H*_3_CH_2_-), 1.30 (2H, sext, *J* = 7.4 Hz, CH_3_C*H*_2_CH_2_-), 1.273 (2H, quint, *J* = 7.4 Hz, -CH_2_C*H*_2_CH_2_N-), 3.48–3.50 (2H, m, =CHC*H*_2_Ar), 3.90 (2H, t, *J* = 7.4 Hz, -NC*H*_2_CH_2_-), 4.40–4.44 (2H, m, -OC*H*_2_CH), 5.82–5.91 (2H, m, 2 × -C*H*=CH-), 7.14 (1H, s, pyrazole-H); ^13^C-NMR (100 MHz, CDCl_3_) δ 13.7, 19.8, 25.7, 32.2, 49.4, 68.2, 125.9, 126.3, 128.3, 128.7, 143.9; HREIMS *m*/*z* calcd. for C_11_H_16_N_2_O [M]^+^ 192.1263, found 192.1264. * Compound **6d** is so unstable to isomerize at room temperature in a couple of days.

Inseparable mixture of *1-n-butyl-7,8-dihydro-1H-oxepino[3,2-c] pyrazole* (**9d**) and *1-n-butyl-5,6-dihydro-1H-oxepino[3,2-c]pyrazole* (**10d**): Oil; IR (film) v_max_ 1654 (C=C), 1565 (C=C), 1492 (C=C) cm^−1^; HREIMS m/z calcd. for C_26_H_23_N_2_O [M]^+^ 192.1263, found 192.1262; **9d**: ^1^H-NMR (600 MHz, CDCl_3_) δ 1.26 (3H, t, *J* = 7.3 Hz, CH_3_CH_2_-), 1.70–1.78 (2H, m, -CH_2_CH_2_N-), 2.42–2.46 (2H, m, =CHCH_2_CH_2_-), 2.87–2.90 (2H, m, ArCH_2_CH_2_-), 3.98 (2H, t, *J* = 7.3 Hz, -NCH_2_CH_2_-), 4.89 (2H, br q. *J* = 7.3 Hz, -OCH=CHCH_2_-), 6.31 (1H, d, *J* = 7.3 Hz, -OCH=CH-), 7.19 (1H, s, pyrazole-H); ^13^C-NMR (150 MHz, CDCl_3_) δ 14.2, 19.1, 23.4, 24.9, 43.3, 60.4, 106.6, 126.5, 127.6, 143.4, 144.4; **10d**: ^1^H-NMR (600 MHz, CDCl_3_) δ 0.93 (3H, t, *J* = 7.6 Hz, CH_3_CH_2_-), 1.11–1.38 (2H, m, CH_3_CH_2_CH_2_-), 1.70–1.78 (2H, m, -CH_2_CH_2_N-), 2.70 (2H, dddd, *J* = 9.1, 5.3, 1.4 Hz, =CHCH_2_CH_2_-), 4.05 (2H, t, *J* = 7.3 Hz, -NCH_2_CH_2_-), 4.13 ( 2H, br dd, *J* = 4.6, 4.4 Hz, -OCH_2_CH_2_-), 5.87 (1H, dt, *J* = 11.2, 5.5 Hz, -CH_2_CH=CH-), 6.35 (1H, dd, *J* =11.2, 0.9 Hz, ArCH=CH-), 7.14 (1H, s, pyrazole-H); ^13^C-NMR (150 MHz, CDCl_3_) δ 13.7, 19.9, 32,5, 34.2, 49.7, 68.5, 116.3, 126.8, 128.5, 143.4, 144.4.

*1-Benzyl-5,8-dihydro-8-methyl-1H-oxepino[3,2-c]pyrazole* (**6h**): Oil; IR (film) v_max_ 1583 (C=C), 1496 (C=C), 1456 (C=C) cm^−1^; ^1^H-NMR (400 MHz, CDCl_3_) δ 1.21 (3H, d, *J* = 7.0 Hz, 8-Me), 3.41 (1H, br quint, *J* = 6.8 Hz, ArC*H* (CH_3_)CH=), 4.36 (1H, dd, *J* = 15.3, 1.9 Hz, -OC*H*HCH=), 4.50 (1H, dd, *J* = 15.3, 5.7 Hz, -OCH*H*CH=), 5.19 (1H, d, *J* = 6.3 Hz, -NC*H*HPh), 5.24 (1H, d, *J* = 6.3 Hz, -NCH*H*Ph), 5.67 (1H, br dt, *J* = 12.0, 5.0 Hz, -CH=C*H*CH_2_-), 5.77 (1H, dd, *J* = 12.0, 6.4 Hz, -CH=C*H*CH-), 7.07 (2H, br d, *J* = 7.4 Hz, Ph-H), 7.23-7.33 (4H, m, Ph-H, pyr-H); ^13^C-NMR (100 MHz, CDCl_3_) δ 21.9, 31.6, 53.5, 68.3, 126.5, 126.7, 127.6, 128.7, 129.4, 131.6, 133.4, 137.1, 142.7; HREIMS *m*/*z* calcd. for C_15_H_16_N_2_O [M]^+^ 240.1263, found 240.1264.

*1-Benzyl-7,8-dihydro-8-methyl-1H-oxepino[3,2-c]pyrazole* (**9h**): Oil; IR (liquid film) v_max_ 1654 (C=C), 1578 (C=C) cm^−1^; ^1^H-NMR (600 MHz, CDCl_3_) δ 1.15 (3H, d, *J* = 7.3 Hz, 8-CH_3_), 2.03 (1H, ddd, *J* = 15.0, 8.8, 3.5 Hz, =CHC*H*HCH-), 2.54 (1H, ddd, *J* = 15.3, 6.4, 2.9 Hz, -=CHCH*H*CH-), 3.14–3.1 (1H, m, -CH_2_C*H*(CH_3_)Ar), 4.71 (1H, dddd, *J* = 7.4, 6.4 Hz, -CH=C*H*CH_2_-), 5.22 (1H, d, *J* = 16.2 Hz, ArC*H*HPh), 5.29 (1H, d, *J* =15.9 Hz, ArCH*H*Ph), 6.29 (1H, dd, *J* = 7.3, 2.6 Hz, -OC*H*=CH-), 7.05 (2H, br d, *J* = 7.0 Hz, Ph-H), 7.24–7.327 (4H, s, Ph-H, pyr-H); ^13^C-NMR (150 MHz, CDCl_3_) δ 20.9, 30.4, 30.7, 53.9, 103.2, 126.4, 127.6, 128.5, 128.7, 132.7, 137.6, 139.9, 142.2; HREIMS *m*/*z* calcd. for C_15_H_16_N_2_O [M]^+^ 240.1263, found 240.1267.

*1-Benzyl-5,6-dihydro-8-methyl-1H-oxepino[3,2-c]pyrazole* (**10h**): Oil; IR (liquid film) v_max_ 1551 (C=C), 1496 (C=C) cm^−1^; ^1^H-NMR (600 MHz, CDCl_3_) δ 2.05 (3H, q, *J* = 1.2 Hz, 8-CH_3_), 2.40–2.44 (2H, m, -CH_2_C*H*_2_CH=), 4.13 (2H, br dd, *J* = 6.6, 4.8 Hz, -CH_2_C*H*_2_O-), 5.45 (2H, br s, NC*H*_2_Ph), 5.77 (1H, tq, *J* = 6.4, 1.2 Hz, -C(CH_3_)=C*H*CH_2_-), 6.95 (2H, br d, *J* = 7.1 Hz, Ph-H), 7.21–7.31 (3H, m, Ph-H), 7.30 (1H, s, pyr-H); ^13^C-NMR (150 MHz, CDCl_3_) δ 22.2, 30.3, 56.2, 73.8, 125.8, 126.4, 126.5, 127.3, 128.6, 129.7, 129.8, 138.2, 143.5; HREIMS *m*/*z* calcd. for C_15_H_16_N_2_O [M]^+^ 240.1263, found 240.1267.

*1-Benzyl-5,8-dihydro-8-phenyl-1H-oxepino[3,2-c]pyrazole* (**6j**): Oil; IR (film) v_max_ 1585 (C=C), 1495 (C=C), 1455 (C=C) cm^−1^; ^1^H-NMR (400 MHz, CDCl_3_) δ(400 MHz, CDCl_3_) δ 4.41 (1H, ddt, *J* = 15.0, 4.1, 1.5 Hz, -OC*H*HCH=), 4.51 (1H, d, *J* = 4.9 Hz, ArC*H*PhCH=), 4.52 (1H, br dd, *J* = 15.0, 5.6 Hz, -OCH*H*CH=), 4.80 (1H, d, *J* = 16.0 Hz, NC*H*HPh), 5.13 (1H, d, *J* = 16.0 Hz, NCH*H*Ph), 5.70 (1H, dddd, *J* = 12.0, 5.1, 3.5, 0.8 Hz, -OCH_2_C*H*=CH-), 5.79 (1H, br dd, *J* = 12.0, 6.0 Hz, -CH=C*H*CHPh), 6.92 (2H, br d, *J* = 8.0 Hz, Ph-H), 7.12–7.39 (8H, m, Ph-H), 7.32 (1H, s, pyr-H); ^13^C-NMR (100 MHz, CDCl_3_) δ 43.6, 53.8, 68.2, 126.3, 126.5, 127.1, 127.5, 127.6, 128.7, 128.7, 129.4, 129.9, 131.0, 136.7, 141.2, 144.5; HREIMS *m*/*z* calcd. for C_20_H_18_N_2_O [M]^+^ 302.1419, found 302.1412. 

*1-Benzyl-7,8-dihydro-8-phenyl-1H-oxepino[3,2-c]pyrazole* (**9j**): Oil; IR (liquid film) v_max_ 1653 (C=C), 1577 (C=C), 1559 (C=C) cm^−1^; ^1^H-NMR (400 MHz, CDCl_3_) δ 2.33 (1H, ddd, *J* = 14.2, 9.2, 4.1 Hz, -CHC*H*HCH=), 2.78–2.84 (1H, m, -CHC*H*HCH=), 4.25 (1H, br t, *J* = 3.9 Hz, ArC*H*(Ph)CH_2_-), 4.67 (1H, ddd, *J* = 8.8, 6.9, 4.9 Hz, -OCH=C*H*CH_2_-), 4.75 (1H, d, *J* = 16.0 Hz, NC*H*HPh), 5.15 (1H, d, *J* = 16.0 Hz, NCH*H*Ph), 6.43 (1H, dd, *J* = 6.7, 2.2 Hz, -OC*H*=CH-), 6.93 (2H, br d, *J* = 5.7 Hz, Ph-H), 7.07 (2H, br d, *J* = 6.5 Hz, Ph-H), 7.20–7.39 (6H, m, Ph-H), 7.39 (1H, s, pyr-H); HREIMS *m*/*z* calcd. for C_20_H_18_N_2_O [M]^+^ 302.1419, found 302.1415. ^13^C-NMR spectrum of **9j** could not be measured due to poor amount of the material.

*5,8-Dihydro-2H-2-trityloxepino[3,2-c]pyrazole* (**7a**); White powder (CH_2_Cl_2_); m.p. 42–48 °C; IR (film) v_max_ 1670 (C=C), 1494 (C=C), 1446 (C=C) cm^−1^; ^1^H-NMR (600 MHz, CDCl_3_) δ 3.60 (2H, m, =CHC*H*_2_Ar), 4.47 (2H, m, 4.7 Hz, -OC*H*_2_CH=), 5.80 (1H, m, -CH=C*H*CH_2_Ar), 6.00 (1H, m, -OCH_2_C*H*=CH-), 6.98 (1H, s, py-H), 7.14–7.18 (6H, m, Tr-H), 7.27–7.34 (9H, m, Tr-H); ^13^C-NMR (150 MHz, CDCl_3_) δ 27.8, 68.6, 78.6, 121.5, 127.1, 127.56, 127.62, 129.5, 130.1, 140.1, 142.6, 143.3; HREIMS *m*/*z* calcd. for C_26_H_22_N_2_O [M]^+^ 378.1732, found 378.1730.

*7,8-Dihydro-2H-2-trityloxepino[3,2-c]pyrazole* (**11a**): Colorless crystals (CH_2_Cl_2_/hexane); m.p. 120–123 °C; IR (KBr) v_max_ 1650 (C=C), 1506 (C=C), 1492 (C=C), 1445 (C=C) cm^−1^; ^1^H-NMR (600 MHz, CDCl_3_) δ 2.39 (2H, m, -=CHC*H*_2_CH_2_-), 2.97 (2H, m, ArC*H*_2_CH_2_-), 4.80 (1H, dt, *J* = 7.8, 5.9 Hz, -CH=C*H*CH_2_-), 6.21 (1H, br dt, *J* = 7.8, 1.5 Hz, -OC*H*=CHCH_2_-), 7.01 (1H, s, py-H), 7.04–7.08 (6H, m, Tr-H), 7.28–7.31 (9H, m, Tr-H); ^13^C-NMR (150 MHz, CDCl_3_) δ 24.8, 27.8, 78.2, 106.1, 121.3, 127.61, 127.64, 130.2, 139.8, 141.4, 142.0, 143.2; HREIMS *m*/*z* calcd. for C_26_H_22_N_2_O [M]^+^ 379.1732, found 378.1730.

*5,6-Dihydro-2H-2-trityloxepino[3,2-c]pyrazole* (**12a**): Colorless crystals (CH_2_Cl_2_/hexane); m.p. 147–151 °C; IR (KBr) v_max_ 1565 (C=C), 1492 (C=C), 1445 (C=C) cm^−1^; ^1^H-NMR (600 MHz, CDCl_3_) δ 2.67 (2H, m, =CHC*H*_2_CH_2_-), 4.14 (2H, br t, *J* = 4.7 Hz, -OC*H*_2_CH_2_-), 5.88 (1H, dt, *J* = 11.2, 5.0 Hz, -CH=C*H*CH_2_-), 6.54 (1H, br d, *J* = 11.2 Hz, ArC*H*=CHCH_2_-), 6.95 (1H, d, *J* = 0.6 Hz, py-H), 7.15–7.19 (6H, m, Tr-H), 7.27–7.30 (9H, m, Tr-H); ^13^C-NMR (150 MHz, CDCl_3_) δ 34.1, 69.6, 78.5, 120.3, 123.1, 127.61, 127.65, 128.4, 130.2, 140.1, 142.8, 143.2; HREIMS *m*/*z* calcd. for C_26_H_22_N_2_O [M]^+^ 378.1732, found 378.1728.

*5,8-Dihydro-2H-2-toluenesulfonyloxepino[3,2-c]pyrazole* (**7c**): Oil; IR (film) v_max_ 1670 (C=C), 1494 (C=C), 1446 (C=C) cm^−1^; ^1^H-NMR (CDCl_3_) δ 2.42 (3H, s, Ar-C*H*_3_), 3.56 (2H, dq, *J* = 5.3, 1.8 Hz, =CHC*H*_2_Ar), 4.48 (2H, dq, *J* = 5.3, 1.2 Hz, =CHC*H*_2_O-), 5.80 (1H, dtt, *J* = 10.3, 5.3, 1.8 Hz, -CH=C*H*CH_2_Ar), 5.97 (1H, dtt, *J* = 10.3, 5.0, 1.2 Hz, -OCH_2_C*H*=CH-), 7.30 (2H, br d, *J* = 8.6 Hz, Ar-*H*), 7.66 (1H, s, py-H), 7.84 (2H, br d, *J* = 8.2 Hz, Ar-*H*); ^13^C-NMR (CDCl_3_) δ 21.7, 27.2, 68.1, 118.4, 127.3, 127.9, 128.6, 129.9, 134.3, 145.5, 145.8, 148.2; HREIMS *m*/*z* calcd. for C_14_H_14_N_2_O_3_S [M]^+^ 290.0725, found 290.0723.

*Dihydro-2H-2-toluenesulfonyloxepino[3,2-c]pyrazole* (**11c**): White power; m.p. 98–102 °C; IR (KBr) v_max_ 1657 (C=C), 1589 (C=C) cm^−1^; ^1^H-NMR (CDCl_3_) δ2.31–2.34 (2H, m, -CH_2_C*H*_2_CH_2_-), 2.42 (3H, s, Ar-C*H*_3_), 2.93–2.96 (2H, m, -CH_2_C*H*_2_Ar), 4.84 (1H, dt, *J* = 7.6, 5.9 Hz, -CH=C*H*CH_2_-), 6.20 (1H, dt, *J* = 7.6, 1.5 Hz, -CH=C*H*Ar), 7.33 (2H, br d, *J* = 8.5 Hz, Ar-*H*), 7.72 (1H, s, py-H), 7.85 (2H, br d, *J* = 8.5 Hz, Ar-*H*); ^13^C-NMR (100 Hz, CDCl_3_) δ 21.7, 23.6, 27.8, 106.7, 118.2, 128.0, 130.0, 134.1, 141.6, 143.3, 145.6, 149.4; HREIMS *m*/*z* calcd. for C_14_H_14_N_2_O_3_S [M]^+^ 290.0725, found 290.0722.

*5,6-Dihydro-2H-2-toluenesulfonyloxepino[3,2-c]pyrazole* (**12c**): White power; m.p. 114–117 °C; IR (KBr) v_max_ 1587 (C=C), 1481 (C=C) cm^−1^; ^1^H-NMR (CDCl_3_) δ 2.41 (3H, s, Ar-C*H*_3_), 2.64–2.68 (2H, m, -CH_2_C*H*_2_CH_2_-), 4.11 (2H, br t, *J* = 14.7 Hz, -CH_2_C*H*_2_O-), 6.10 (1H, dt, *J* = 11.4, 5.0 Hz, -CH=C*H*CH_2_-), 6.51 (1H, br d, *J* = 11.4, Hz, ArC*H*=CH-), 7.31 (2H, br d, *J* = 8.2 Hz, Ar-*H*), 7.63 (1H, d, *J* = 0.8 Hz, py-H), 7.85 (2H, br d, *J* = 8.2 Hz, Ar-*H*); ^13^C-NMR (CDCl_3_) δ 21.7, 33.9, 69.8, 117.1, 121.7, 128.0, 129.9, 128.6, 129.9, 133.7, 145.2, 147.6; HREIMS *m*/*z* calcd. for C_14_H_14_N_2_O_3_S [M]^+^ 290.0725, found 290.0725.

### 3.6. Double-Bond Migration of 5,8-Dihydro-1H-1-trityloxepino[3,2-c]pyrazole (***6a***) Catalyzed by Ruthenium Hydride Species

To a CH_2_Cl_2_ solution (4 mL) of **6a** (9.6 mg, 0.025 mmol) in a MW vial (2–5 mL), RuClH(CO)(PPh_3_)_3_ (2.6 mg, 0.0027 mmol) was added. The vial was sealed and heated under MW irradiation at 140 °C for 10 min. The cooled reaction mixture was evaporated under reduced pressure. The residue was purified by preparative TLC (eluent: hexane/EtOAc = 10:1 *v*/*v*), affording **9a** (4.3 mg, 45%) and **10a** (1.2 mg, 13%).

## 4. Conclusions

A new method was developed for constructing a heterocyclic system, dihydro-1*H*- or 2*H*-oxepino[3,2-*c*]pyrazoles, from 4-hydroxy-1*H*-pyrazoles via Claisen rearrangement and RCM as the key steps. The 2nd generation Grubbs catalyst was proved to be a suitable catalyst for the RCM. The RCM reactions of 3- or 5-allyl-4-allyloxy-1*H*-pyrazoles at room temperature gave the normal RCM products in good yields. However, the RCM reactions under MW irradiation at a high temperature afforded the RCM products with double-bond rearrangement. The method described in this study provides diverse dihydrooxepino[3,2-*c*]pyrazoles depending on the reaction conditions. Using a similar strategy with RCM, syntheses of novel pyrazole-fused heterocycles are ongoing. Bioactivities, such as COX-inhibition or glycosidase inhibition, of synthesized compounds might be evaluated in our following studies.

## Figures and Tables

**Table 1:**
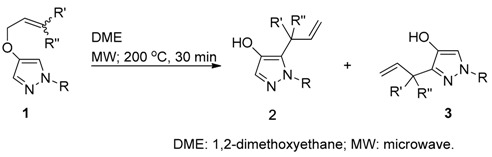
Regioselectivity of Claisen rearrangement of **1**.

Entry	Substrate	R	R’	R’’	Product, Yield (%)	
1 ^a^	**1a**	Tr	H	H	**2a** (65)	**3a** (1)
2	**1b**	Bn	H	H	**2b** (98)	**3b** (0)
3	**1c**	Ts	H	H	**2c** (0)	**3c** (55)
4	**1d**	*n*-Bu	H	H	**2d** (97)	**3d** (0)
5 ^b^	**1e**	Tr	Me	H	**2e** (0)	**3e** (0)
6 ^b^	**1f**	Tr	Me	Me	**2f** (0)	**3f** (0)
7 ^b^	**1g**	Tr	Ph	H	**2g** (0)	**3g** (0)
8	**1h**	Bn	Me	H	**2h** (64)	**3h** (0)
9 ^b^	**1i**	Bn	Me	Me	**2i** (0)	**3i** (0)
10	**1j**	Bn	Ph	H	**2j** (54)	**3i** (0)

^a^ Reference [13,14]; ^b^ no reaction.

**Table 2:**
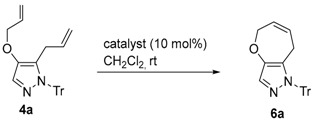
Ring-closing metathesis (RCM) of **4a** with various Grubbs catalysts.

Entry	Catalyst	Time (min)	6a, Yield (%)
1	Grubbs^1st^	150	47
2	Grubbs^2nd^	120	74
3	Hoveyda-Grubbs^2nd^	150	66

**Table 3:**
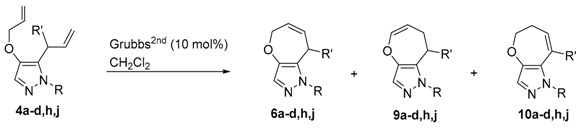
RCM of 1-substituted 4-allyloxy-5-allyl-1*H*-pyrazoles.

Entry	Substrate	R	R’	Temperature (°C)	Time (min)	Product, Yield (%)		
1	**4a**	Tr	H	40 (reflux)	overnight	**6a** (0)	**9a** (27)	**10a** (41)
2	**4a**			140 (MW)	1	**6a** (4)	**9a** (23)	**10a** (27)
3	**4a**			140 (MW)	2	**6a** (8)	**9a** (30)	**10a** (18)
4	**4a**			140 (MW)	5	**6a** (11)	**9a** (38)	**10a** (29)
5	**4a**			140 (MW)	10	**6a** (5)	**9a** (41)	**10a** (33)
6	**4b**	Bn	H	r.t.	120	**6b** (63)	**9b** (0)	**10b** (4)
7	**4b**			140 (MW)	10	**6b** (0)	**9b** (25)	**10b** (63)
8	**4c**	Ts	H	r.t.	120	**6c** (70)	**9c** (0)	**10c** (0)
9	**4c**			140 (MW)	10	**6c** (0)	**9c** (21)	**10c** (50)
10	**4d**	*n*-Bu	H	r.t.	120	**6d** (38)	**9d** (4) ^a^	**10d** (18) ^a^
11	**4d**			r.t.	60	**6d** (76)	**9d** (trace)	**10d** (trace)
12	**4d**			140 (MW)	10	**6d** (0)	**9d** (19) ^a^	**10d** (58) ^a^
13	**4h**	Bn	Me	r.t.	120	**6h** (87)	**9h** (0)	**10h** (0)
14	**4h**			140 (MW)	10	**6h** (48)	**9h** (6)	**10h** (21)
15	**4j**	Bn	Ph	r.t.	120	**6j** (73)	**9j** (0)	**10j** (0)
16	**4j**			140 (MW)	10	**6j** (70)	**9j** (trace)	**10j** (0)

^a^ inseparable, calculated from ^1^H-NMR spectra of the mixture; r.t.: room temperature.

**Table 4:**
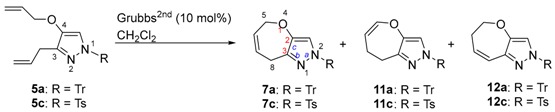
RCM of 1-substituted 4-allyloxy-3-allyl-1*H*-pyrazoles.

Entry	Substrate	Temperature (°C)	Time (min)	Product, Yield (%)		
1	**5a**	rt	120	**7a** (75)	**11a** (0)	**12a** (0)
2	**5a**	140 (MW)	10	**7a** (0)	**11a** (13)	**12a** (79)
3	**5c**	rt	120	**7c** (52)	**11c** (0)	**12c** (0)
4	**5c**	140 (MW)	10	**7c** (trace)	**11c** (11)	**12c** (56)
